# Supplementary material for: Novel Variants of SCCmec Type IX Identified in Clonal Complex 398 Livestock-Associated Methicillin-Resistant Staphylococcus aureus from Pork Production Systems in Korea
Source: Antibiotics (Basel). 2025 Feb 21;14(3):217. doi: 10.3390/antibiotics14030217 (PMC11939326; doi:10.3390/antibiotics14030217)
Supplement: Supplementary file 1 [file antibiotics-14-00217-s001.zip › antibiotics-3478333-supplementary.pdf]

Table S1. Genomic characteristics of the KF1A-1172 and JS1E-122 strains

Strain	KF1A-1172	JS1E-122
Total length (bp)	2,887,931	2,901,962
GC content (%)	32.9	33.0
Coverage	330X	167X
ORFs	2,699	2,721
tRNA	59	59
rRNA	19	19
No. of plasmid (size)	4	2
	Contig2 (56206 bp),<br>Contig3 (10473 bp),<br>Contig4 (2992 bp),<br>Contig5 (2366 bp)	Contig2 (4565 bp),<br>Contig3 (2555 bp)

Table S2. Antimicrobial and heavy metal resistance genes of KF1A-1172

Genes	% identity	Query/template length	Position in contig (No.)	Protein function
**Aminoglycosides**				
aadD	100	762/762	1937..2698 (3)	Aminoglycoside resistance
aac(6′)-*aph*(2′′)	100	1440/1440	1354238..1355677 (1)	Aminoglycoside resistance
	100	1440/1440	39200...40639 (2)	
ant(6)-la	100	864/864	50146..51009 (2)	Aminoglycoside resistance
bleO	100	316/316	2921..3236 (3)	Aminoglycoside resistance
	100	104/104	8373..8476 (3)	
**Beta-lactams**				
mecA	100	2007/2007	2494889..2496894 (1)	Beta-lactam resistance
blaZ	99.88	845/846	1647125..1647970 (1)	Beta-lactam resistance
**MLS - Macrolides, Lincosamides and Streptogramins**				
erm(C)	100	735/735	742..1476 (5)	Macrolide resistance
lsa(E)	100	1485/1485	45083..46567 (2)	Lincosamide resistance
lnu(B)	100	804/804	44226..45029 (2)	Lincosamide resistance
**Phenicols**				
fexA	99.65	1423/1428	1349820..1351247 (1)	Phenicol resistance
**Tetracyclines**				
tet(M)	100	1920/1920	594685..596604 (1)	Tetracycline resistance
**Trimethoprims**				
dfrE	100	492/492	7185..7571 (3)	Trimethoprim resistance
**Quinolones**				
gyrA(S84A)	99	2663/2664	2417788..2420457 (1)	Quinolone resistance
parC(S80Y)	99	2402/2403	1020611..1023013 (1)	Quinolone resistance
**Heavy metal**				
czrC	100	1926/1926	2505629..2507554 (1)	Pb/Cd/Zn antiporter
czcD	100	867/867	2083620...2084486 (1)	Cd/Co/Zn antiporter
	100	960/960	2615811..2616770 (1)	
cadD	100	618/618	19084..19701 (1)	Cadmium transporter

Table S3. Antimicrobial and heavy metal resistance genes of JS1E-122

Genes	% identity	Query/template length	Position in contig (No.)	Protein function
Aminoglycosides				
aadD	100	762/762	1250703..1251464 (1)	Aminoglycoside resistance
aac(*6′*)-*aph*(*2″*)	100	1440/1440	1277703..1279142 (1)	Aminoglycoside resistance
ant(6)-la	100	864/864	1261199..1262062 (1)	Aminoglycoside resistance
str	100	849/849	701..1549 (2)	Aminoglycoside resistance
Beta-lactams				
mecA	100	2007/2007	1608489..1610494 (1)	Beta-lactam resistance
blaZ	99.89	887/888	2424690..2425577 (1)	Beta-lactam resistance
	100	849/849	1224795..1225643 (1)	
MLS - Macrolides, Lincosamides and Streptogramins				
erm(T)	100	735/735	1268280..1269014 (1)	Macrolide resistance
lsa(E)	100	1485/1485	1256136..1257620 (1)	Lincosamide resistance
lnu(B)	100	804/804	1255279..1256082 (1)	Lincosamide resistance
Phenicols				
fexA	99.79	1425/1428	2716751.. 2718178 (1)	Phenicol resistance
Tetracyclines				
tet(M)	100	1920/1920	623832..625751 (1)	Tetracycline resistance
tet(L)	100	1377/1377	1269658..1271034 (1)	Tetracycline resistance
Trimethoprims				
dfrG	100	498/498	1282659..1283156 (1)	Trimethoprim resistance
	100	498/498	1221556..1222053 (1)	
Quinolones				
gyrA(S84L)	99	2663/2664	1647236..1649899 (1)	Quinolone resistance
parC(S80Y)	99	2402/2403	196029..198431 (1)	Quinolone resistance
Heavy metal				
czrC	100	1926/1926	1598229..1600154 (1)	Pb/Cd/Zn antiporter
czcD	100	960/960	1463934..1464893 (1)	Cd/Co/Zn antiporter
	100	981/981	2269869..2270849 (1)	
cadC	100	348/348	1241844..1242191 (1)	Cadmium transporter
